# Can electronic assessment tools improve the process of shared decision-making? A systematic review

**DOI:** 10.1177/1833358320954385

**Published:** 2020-10-05

**Authors:** Nyantara Wickramasekera, Sarah K Taylor, Elizabeth Lumley, Thomas Gray, Emma Wilson, Stephen Radley

**Affiliations:** 1 7315 University of Sheffield, UK; 2 5994 Newcastle University, UK; 3 7318 Sheffield Teaching Hospitals NHSFT, UK

**Keywords:** decision-making, organisational, e-health, electronic clinical documentation, patient safety, review, systematic, health information technology, health information management

## Abstract

**Background:**

Patient involvement in decision-making plays a prominent role in improving the quality of healthcare. Despite this, shared decision-making is not routinely implemented. However, electronic assessment tools that capture patients’ history, symptoms, opinions and values prior to their medical appointment are used by healthcare professionals during patient consultations to facilitate shared decision-making.

**Objective:**

To assess the effectiveness of electronic assessment tools to improve the shared decision-making process.

**Method:**

A systematic review was conducted following PRISMA guidelines. Published literature was searched on MEDLINE, EMBASE and PsycINFO to identify potentially relevant studies. Data were extracted and analysed narratively.

**Results:**

Seventeen articles, representing 4004 participants, were included in this review. The main findings were significant improvement in patient–provider communication and provider management of patient condition in the intervention group compared to the control group. In contrast, patient–provider satisfaction and time efficiency were assessed by relatively few included studies, and the effects of these outcomes were inconclusive.

**Conclusion:**

This review found that communication and healthcare professional’s management of a patient’s condition improves because of the use of electronic questionnaires. This is encouraging because the process of shared decision-making is reliant on high-quality communication between healthcare professionals and patients.

**Implications:**

We found that this intervention is especially important for people with chronic diseases, as they need to establish a long-term relationship with their healthcare provider and agree to a treatment plan that aligns with their values. More rigorous research with validated instruments is required.

## Introduction

Shared decision-making is an important aspect of healthcare delivery, which is associated with improved health outcomes, patient satisfaction and reduced costs (Brackett and Kearing, 2015; Carpenter et al., 2011; Knops et al., 2013; NHS England, 2014; O’Connor et al., 2003; Sawesi et al., 2016; Stacey et al., 2011; Vlemmix et al., 2013). According to the definition of shared decision-making, the process involves “clinicians and patients working together to select tests, treatments, management or support packages; based on clinical evidence and the patient’s informed preferences” (Coulter and Collins, 2011). The treatment and management of many conditions often includes consideration of a number of different options, including weighing up any corresponding advantages and drawbacks of potential interventions or treatment strategies. It is uncommon for a single “standard” care option to exist, this being defined as “the option which is almost universally recognised by patients as being the most desirable choice of treatment” (Barry and Edgman-Levitan, 2012). Typically, the healthcare professional is able to present and discuss the available options, including no further treatments or intervention, alongside their potential benefits and/or disadvantages. The patient provides his or her opinions and a psychosocial context. Both may be considered “experts” in their individual areas (Coulter, 1999). Only by acknowledging this, and working together, can this information be used by both parties to reach a decision that aligns with the patient’s values. This new model of respecting patients’ opinions, and involving them in their own healthcare planning, is in direct contrast to the previous paternalistic model of decision-making.

The importance of shared decision-making to clinical practice is widely acknowledged (Corrigan, 2005; Lansley, 2010; Right Care, 2018; Wohlgemuth et al., 2019). Global research data show that there is large geographical variation in the number of surgical procedures carried out for the same conditions, particularly in the United States (Wennberg et al., 2007). This variation may suggest that some patients undergo unnecessary investigations and treatments, with the associated exposure to risk and harm, as well as waste of resources (Epstein et al., 2005). Moreover, poor adherence to medication, missed opportunities to manage conditions and legal disputes can arise from poor communication around decisions about care (Harrison and Memel, 1994; Vermeire et al., 2001; Zolnierek and DiMatteo, 2009). For example, the landmark UK Supreme Court medical negligence case (*Montgomery v Lanarkshire Health Board*) ruled that doctors should seek informed consent only when patients are fully informed about the risks and benefits of all reasonable options including no treatment (Campbell, 2015). Furthermore, in the United Kingdom, a series of official inquiries (i.e. Mid Staffordshire, Morecambe Bay and Bristol) into poor patient treatment and care have recommended that the “patient voice should be heard and heeded at all times” to reduce the risk of harm to patients (Francis, 2010, 2013; Kennedy, 2001; Kirkup, 2015). Consequently, engaging patients in the decision-making process is vitally important to the delivery of high-quality healthcare and to reduce risk and empower patients.

Different interventions are utilised by healthcare professionals to improve the shared decision-making model, and these can include electronic assessment tools and decision aids. Electronic assessment tools are computerised questionnaires, used to capture patients’ history, symptoms, opinions and values that are used during patient consultations. Decision aids provide information to patients about the treatment options available to them in an easy to understand manner and help patients to select a single treatment option that aligns with their values (Brackett and Kearing, 2015; Stacey et al., 2011). Up-to-date evidence from systematic reviews has shown that decision aids can reduce decisional conflict and increase patient understanding of treatment options (Carpenter et al., 2011; Knops et al., 2013; O’Connor et al., 2003; Stacey et al., 2011; Vlemmix et al., 2013). While these interventions are important to aid shared decision-making, these tools are confined to the final decision-making stage of the treatment process. Consequently, to engage patients and clinicians, electronic assessment tools are increasingly used at the early stages of the treatment process to improve the efficiency and effectiveness of clinical assessment and consultation (Ruland et al., 2003). It is hypothesised that the provision of an objective and comprehensive patient history, including their personal perspective, opinions and values to the healthcare professional, can enable both parties to work together to select the most appropriate strategy to treat or manage the condition, aligning patient values with patient choice (Hargraves et al., 2016; Ruland et al., 2003). To date, systematic review evidence about the role of electronic assessment tools in this process has not been published. This systematic review assessed the effectiveness of electronic assessment tools to improve the shared decision-making process.

## Method

### Search strategy

This systematic review was conducted following PRISMA guidelines, which provide rigorous standards for conducting healthcare reviews (Moher et al., 2009). A comprehensive search was conducted in MEDLINE, EMBASE and PsycINFO to identify potentially relevant studies. The initial search was conducted in 2017, subsequently the search was rerun in 2020. Studies were also sought by manually searching bibliographies of included studies to ensure that the search was comprehensive. Searches were designed, with the help of an information specialist, to identify electronic clinical assessment tools by combining the search concepts “electronic clinical assessment tools” and “shared decision-making” (see Online Supplementary Material). Due to the limited availability of resources, we excluded non-English language publications. Search strategies were similar across databases, but where necessary search syntax was adapted for the individual platform.

### Study selection

Studies were included if an electronic clinical assessment tool was completed by the patient prior to a patient–provider consultation and the impact this had on decision-making was evaluated. Paper-based questionnaires or decision aids (where patients are given information about the effectiveness or safety of available treatment options) were excluded because these were either not designed to promote shared decision-making or have been extensively evaluated to date (Carpenter et al., 2011; Knops et al., 2013; O’Connor et al., 2003; Stacey et al., 2011; Vlemmix et al., 2013). Studies involving all types of patients and providers were included in the review, including in primary care and secondary care settings. Studies evaluating the feasibility of implementing electronic questionnaires were excluded. Only quantitative studies were included as initial scoping searches failed to identify sufficient qualitative studies. Thus, qualitative studies, systematic reviews, conference abstracts, opinion papers, editorials and protocols were excluded. Studies were included if they evaluated one of the following outcome measures: shared decision-making, patient–provider communication, patient or provider satisfaction, patient participation, reduce decisional conflict and patient–doctor interaction.

### Data extraction

Titles and abstracts were screened independently by two reviewers (NW and SKT/EL) using the inclusion criteria listed above. Full paper copies of potentially relevant studies were retrieved and independently assessed by two reviewers (NW and SKT/EL). At each screening stage, disagreements were resolved by consensus. The number of studies included or excluded at the two-stage screening process was recorded in a PRISMA diagram. EndNote X9 software was used to manage references during the screening stage. A pilot tested data extraction form was filled for each included study by one reviewer (NW) and checked by a second reviewer (SKT).

### Assessment of risk of bias

To ensure internal validity of the systematic review, a risk of bias analysis was conducted. Cochrane Collaborations “suggested risk of bias criteria for EPOC reviews” was used to assess the risk of bias of included studies (Higgins and Green, 2011). The studies with all components rated “low risk” indicated that studies were free from systematic error; “high risk” represented that studies were not free from systematic error; and “unclear risk” represented insufficient data were reported to make a conclusive judgement.

### Data synthesis

Due to insufficient homogeneity in the studies, conducting meta-analyses was not deemed appropriate and data were therefore synthesised narratively, including a description of study design, setting, population, intervention and outcomes for each included study. The results for each outcome were analysed according to two dimensions: (1) whether results showed a significant positive or negative effect and (2) whether there was consistency in the effects for each individual outcome across the included studies. In addition, an organising framework was used to analyse the results of the outcome category called “patient–provider communication” (Roter, 2000). This framework was useful to separate this large outcome category into smaller conceptually distinct communication elements.

## Results

### Search results

A total of 1622 citations were identified in the initial searches of published studies; the rerun of the search in 2020 identified a further 668 citations. After duplicates were removed, 1846 references were screened, based on title and abstract. Subsequently, 128 potentially relevant articles were reviewed in full; of these, 17 papers were included, which arose from 15 trials (the results of one trial were published in three papers). The remaining 111 papers were excluded as they did not meet the inclusion criteria. For example, these studies evaluated electronic tools that were not used during a consultation (*n* = 36); used paper-based questionnaires (*n* = 18); evaluated the feasibility of implementing electronic assessments (*n* = 17); and evaluated the effectiveness of a decision aid, rather than an assessment tool (*n* = 7) and virtual clinics (*n* = 9). The results of the search further detailing the inclusion exclusion process can be found in the PRISMA diagram (see Online Supplementary Material).

### Study characteristics

Eight of the 15 trials were randomised controlled trials, three were quasi-experimental trials, three were cohort studies and one was a non-randomised pilot study (see Table 1). Sample sizes ranged from 52 (Ruland et al., 2003) to 1281 (Rhodes et al., 2006), with most studies between 120 and 288 participants. A total of 4004 participants were included in this systematic review. These studies were conducted in seven high-income countries. Six (40%) studies were conducted in the United States, followed by two (13%) each from the Netherlands and Norway and one study from the United Kingdom, Australia, Germany, Denmark and Canada (see Table 1).

**Table 1.** Characteristics of included studies.

Reference	Study design	Country and setting	Population	Total number of participants	Description of intervention	Description of control group
Barnabel et al. (2008)	RCT	USA, Tertiary care centre (obstetrics and gynaecology)	Women with an appointment to see a gynaecologist	288	The TalkToYourDoc (TTYD) tool was filled by participants and answered customised questions about medical history, drug prescriptions, and severity of menopausal symptoms. Pop-up windows were available to explain important terms. A print out was generated, for the patients to take to their healthcare provider.	Usual care (no further details available)
Berry et al. (2011)	RCT	USA, Seattle Cancer Care Alliance or the University of Washington Medical Centre	Adult patients (18 years or older) with a cancer diagnosis starting a new treatment	660	Patients filled the ESRA-C questionnaire on touchscreen computers while waiting for their clinic appointment. They were asked to fill in details about their cancer-related symptoms and quality-of-life issues (SQLIs). This tool produced a graphical summary, which was printed and included in the patients file before the appointment. Also the report flagged up whether the participant’s quality of life issues was above or below a predetermined threshold but no other recommendations were given.	Participants in the control group also completed the ESRA-C questionnaire, but the results were not provided to the clinicians
Boyes et al. (2006)	Non-randomised pilot study	Australia, public cancer treatment centre	Patients vising a medical oncologist for their first consultation	80	Patients completed a survey on touchscreen computers while waiting to see their oncologist. During the survey, patients were asked about their physical symptoms in the past 7 days, level of anxiety and depression, and their care needs. Subsequently, the computer software immediately produced a feedback report, which was printed and attached to the patient’s file for reflection during the consultation. The software suggested strategies to manage each identified problem.	The control group participants also filled the survey but the results were not provided to the oncologist.
Haverman et al. (2013)	Sequential cohort design	Amsterdam, 4 Paediatric Rheumatology clinics	Children aged 0–18 years with juvenile idiopathic arthritis	176	Children (or parents) completed the ePROfile online questionnaire which consisted questions on quality of life, and functional ability. The answers were then automatically converted and colour coded to highlight problem areas. The paediatric rheumatologist accessed the patient’s ePROfile directly from the Web site during consultation.	During the control period, the ePROfile was not discussed
Posthuma et al. (2016)	RCT	The Netherlands, Urogynaecology department of a teaching hospital	Women with pelvic organ prolapse or urinary incontinence problems	120	Participants received an email to complete the web-based questionnaire which consisted questions on medical history, medication used, smoking and drinking habits, pelvic functioning (incontinence, sexual functioning etc.), socio-economic and quality of life information. Participants who couldn’t access the Internet at home completed the questionnaire at the hospital. A summary was automatically generated for the gynaecologist	No questionnaire used
Rhodes et al. (2006)	RCT	USA, two Emergency Departments (urban and suburban)	Women aged 18 to 65 years attending EDs	1281	Women completed the survey about domestic violence, in a private room using touchscreen computers. If they were deemed at risk of intimate partner violence, the tool suggested referral options to the clinician. A summary document was printed and included in the patient file to be discussed by the clinician.	Patients did not complete a questionnaire in the control group
Ruland et al. (2003)	RCT	USA, Dartmouth Hitchcock Medical Centre	Cancer patients 21 years and above visiting outpatient clinics	52	CHOICEs intervention was administered on tablet computers at outpatient clinics. The tool collected patient’s cancer-specific symptoms and any functional problems. It also included a shared decision-making component that provided the clinicians information on symptom severity ranked by the importance of problem categories to the patients. CHOICEs also used conditional tailoring, so that questions were tailored based on a patient’s previous response. An assessment summary was included in the patient file to be used during the consultation.	Participants completed CHOICEs but summary reports were provided to the clinician
Ruland et al. (2010)	RCT	Norway, Specialised care and teaching hospital	Adults starting treatment for a newly diagnosed acute myelogenous leukaemia, lymphatic leukaemia, multiple myeloma, Hodgkin disease, or non-Hodgkin lymphoma	161	Patients filled the CHOICE ITPA computerised assessment instrument which gathered information on patient symptoms, problems and concerns. The instrument produced follow-up questions which were tailored to each patient, depending on their initial response. Patients used touchpad tablets to fill the questionnaire. The instrument allowed patients to “prioritise and rank their problems.” The assessment summary was included in the patient’s file to be discussed during the appointment.	The control group filled in the Choice ITPA, but the assessment summaries were not provided to the healthcare professional
Schussler-Fiorenza Rose et al. (2015)	RCT	USA, women’s health internal medicine clinic in Wisconsin	Women aged 40 years or older, who were scheduled for a routine physical appointment at a women’s health clinic	145	The electronic pelvic floor questionnaire (ePAQ-PF) was filled by patients at the clinic 30 min prior to the appointment. It included questions on urinary, bowel, vaginal and sexual functioning. The instrument used an adaptive testing technique to tailor the questionnaire to each patient. The questionnaire asked participants to list the symptoms in order of severity. The summary report was printed and given to the clinician and participant.	Patients in the control group completed ePAQ-PF after their appointment.
Taenzer et al. (2000)	Sequential cohort design	Canada, outpatient lung clinic	Patients with lung cancer at outpatient clinic	53	Patients completed the EORTC QLQ-C30 Questionnaire on a PC in the clinic waiting room. This quality of life questionnaire collected patients’ physical, emotional, cognitive, social and role functions. The computer program produced a summary report of the items that were a priority to the participants, and this report was provided to the clinical staff before the appointment	Participants completed the EORTC QLQ-C30 paper version after the clinic appointment.
Velikova et al. (2004)	RCT	UK, Leeds Cancer Centre Medical Oncology Clinic	Oncology patients starting new treatment at clinic	286	Patients completed questionnaires using touchscreen computers. They completed the EORTC QLQ-C30 Questionnaire, and the Hospital Anxiety and Depression Scale. Subsequently, graphic printouts of results were provided to the physicians to use during appointments.	No touchscreen measurement of questionnaires before the appointment
Heyn el al. (2012, 2013a, 2013b)	Quasi-experimental design	Norway, Outpatient clinics at a University hospital in Oslo	Patients with leukaemia, lymphoma, multiple myeloma or testicular cancer	196	The CHOICE ITPA tool was used to gather patient information such as their symptoms and problems and prioritise problems in the order they needed help from the clinician. This was filled on touchscreen computers and the summary reports were provided to the clinicians or nurses.	Control group received no intervention
Schuler et al. (2017)	Cohort study	Cancer centre at hospital in Germany	Cancer patients	126	An electronic questionnaire was filled by the participants on tablet PCs prior to the appointment. The summary scores were calculated an updated on the hospital information system (HIS) which the physicians had direct access to. The data collected were shown in a visualised and accessible way on the HIS.	No control group
Recinos et al. (2017)	Cohort study	Clinics in the USA	Patients with neurological disorders	323	Patients completed electronic questionnaires on tablets in clinic or at home using the Knowledge Program electronic software platform. Patients completed generic quality of life questions and disease-specific scales. Prior to the appointment, these data were available for the healthcare professional to review.	No control group
Tolsturp et al. (2020)	Clinical trial sub-study (Cohort study)	University hospital in Denmark	Melanoma patients	57	The patients were given a tablet to complete the electronic questionnaire at home once a week. They completed the Common Terminology Criteria for Adverse Events tool. These data were immediately available for the healthcare professionals to view; however, no alters were sent to them. The system colour coded the symptoms depending on the severity as green, yellow or red. The clinicians viewed this report at outpatient clinics.	No control group

RCT: randomised controlled trial; EORTC QLQ-C30: European Organization for Research and Treatment of Cancer Core Quality of Life Questionnaire.

All studies took place in secondary care settings. Populations most often studied were cancer patients (*n* = 9), followed by patients attending gynaecology appointments (*n* = 3); the remaining three studies evaluated patients with neurological disorders, domestic violence at emergency departments and juvenile idiopathic arthritis at paediatric rheumatology clinics (see Table 1). Doctors were the provider of interest in all studies, but in two studies the focus was on both doctors and nurses (Heyn et al., 2013a; Ruland et al., 2010).

### Intervention characteristics

Included studies evaluated 13 different electronic assessment tools (see Table 1). One tool called “Choice” was evaluated three times, once in the United States and twice in Norway (Heyn et al., 2013b; Ruland et al., 2010; Ruland et al., 2003). Intervention components common to many studies included the taking of a patient’s medical history, documenting symptoms related to their condition, quality of life status and personal care needs. In addition, two studies included further questions to improve shared decision-making by asking patients to highlight the symptoms that they consider a priority so that providers could address problems that were considered important to patients (Ruland et al., 2003; Schüssler-Fiorenza Rose et al., 2015). Four studies used instruments tailored to individual respondents; these instruments produced specific follow-up questions depending on the patient’s response to the previous question (Heyn et al., 2012; Ruland et al., 2010; Ruland et al., 2003; Schüssler-Fiorenza Rose et al., 2015).

The majority (10 studies) of interventions were delivered on touchscreen computers or tablets while patients were waiting for their appointment (see Table 1). Subsequently, the summary results of the electronic questionnaires were printed and added to the patient’s charts available for the provider to use during the consultation. Four studies invited patients to complete the electronic questionnaire at home (Barnabei et al., 2008; Haverman et al., 2013; Posthuma et al., 2016; Tolsturp et al., 2020). In these studies, providers accessed the results of the electronic questionnaire directly from the online system (Barnabei et al., 2008; Haverman et al., 2013; Posthuma et al., 2016; Tolsturp et al., 2020), or patients brought the printouts of the results to the consultation (Barnabei et al., 2008). One study gave the patient the option to complete questionnaires on tablets in clinic or at home (Recinos et al., 2017). Most electronic tools only produced summary results of the questionnaires; however, three studies included extra functionality (Berry et al., 2011; Boyes et al., 2006; Rhodes et al., 2006). In two studies, the software suggested strategies or referral options to address the identified issues (Boyes et al., 2006; Rhodes et al., 2006); and another study flagged patients who lay above a predetermined threshold but did not provide recommendations to address the issues flagged (Berry et al., 2011).

### Risk of bias assessment

The evidence arises from 15 trials, of which only four were rated as “low risk” (Barnabei et al., 2008; Posthuma et al., 2016; Rhodes et al., 2006; Ruland et al., 2010), indicating that studies were free from systematic error. The remaining studies were rated as “high risk” or “unclear risk,” which suggests that these studies had many limitations or insufficient information was provided in the article to make a conclusive judgement. A serious limitation of the included studies was the risk of contamination, three studies reported that both arms of the trial were run in parallel in the same clinic, this means that clinicians would see patients from both the intervention and control arms and might inadvertently alter their usual consultation behaviour (Berry et al., 2011; Boyes et al., 2006; Velikova et al., 2004). Also, in six studies, poor reporting was common with studies failing to report whether outcomes were assessed blindly, if allocation was concealed, or if steps were taken to reduce contamination (Berry et al., 2011; Boyes et al., 2006; Haverman et al., 2013; Heyn et al., 2013a; Ruland et al., 2003; Taenzer et al., 2000). Moreover, five studies were rated as “high risk” for selection bias, because the authors used a non-random method of participant selection such as sequentially assigning participants to the intervention or control groups (Boyes et al., 2006; Haverman et al., 2013; Heyn et al., 2013a; Ruland et al., 2003; Taenzer et al., 2000). Furthermore, three studies were rated as “high risk” because there was no control group and thus the results should be interpreted with caution (Recinos et al., 2017; Schuler et al., 2017; Tolsturp et al., 2020).

### Intervention effectiveness

The included studies measured a wide range of primary outcomes; to support data synthesis, results were grouped into four outcome categories.

### Patient–provider communication

*Information giving:* This encompassed patient’s giving information to the healthcare provider and vice versa and is the first and largest subcategory of patient–provider communication (see Table 2). Six studies were included in this category, and of these, four reported significant positive results (Berry et al., 2011; Haverman et al., 2013; Heyn et al., 2013a; Velikova et al., 2004); observing that the electronic tool enhanced information exchange between patient and provider. For example, patients disclosed more quality of life issues, psychosocial problems and cancer-related symptoms. One study reported that clinicians provided more information to the intervention group compared with the control group (Heyn et al., 2013a). However, two studies measuring the disclosure of domestic violence and faecal incontinence found no significant difference in disclosure between the intervention group and the control group (Rhodes et al., 2006; Schüssler-Fiorenza Rose et al., 2015).

**Table 2.** Patient–provider communication.

Outcomes	Definition and instrument	Reference	Results
Subgroup 1: Information giving			
Discussion of symptoms and quality of life issues by clinicians and patients	Authors coded audio recorded communication between clinicians and patients	Berry et al. (2011)	The intervention group discussed significantly more symptoms and quality of life issues (OR = 1.287, 95% CI: 1.047, 1.583) compared with the control condition. Impact of cancer treatment on sexual activities and issues relevant to social functioning were more likely to be discussed in the intervention group
Communication about HRQoL	After every consultation, the paediatric rheumatologists and the parents scored whether the following 12 HRQoL topics were discussed: Condition/Energy, Sports, Autonomy, Pain/Physical Problems, Use of Medicine, Sleep, Eating/Drinking, Emotions, Family, Contact with Peers, School and Cognition, and Attendance at School	Haverman et al. (2013)	Use of the ePROfile significantly increased discussion of psychosocial topics. For example, parents reported that “emotions” and “family” were discussed more often in the intervention group than in the control group. Paediatric rheumatologists reported that “family” and “contact with peers” were more often discussed in the intervention group than in the control group
Discussion of HRQoL issues	Measured by content analysis of tape-recorded physician–patient encounters. A study-specific checklist was used to note whether HRQoL issues included in EORTC QLQ-C30 were discussed	Velikova et al. (2004)	The number of EORTC QLQ-C30 symptoms mentioned during the encounters was higher in the intervention group in comparison with the control group. More frequent discussion of chronic nonspecific symptoms (difficulty sleeping, lack of appetite and fatigue) was observed, without prolonging the encounters
Patient and clinician participation in consultation	Consultation content was coded using a symptom list from the Choice ITPA	Heyn et al. (2013b)	The patients in the intervention group addressed significantly more symptoms compared to the control group (*d* = 0.42, *p* < 0.05). Clinicians provided more information to the intervention group compared to the control group
Faecal incontinence disclosure	Mentioned in the clinic notes, or in the post-visit discussion	Schussler-Fiorenza Rose et al. (2015)	FI mention in the clinic note was rare (only 7 instances in total, 2 of which were newly diagnosed FI) and did not differ between the two groups (pre 3% vs. post 2%, *p* = 0.74)
Domestic violence disclosure	Assessed using previously validated questionnaires: the Abuse Assessment and Partner Violence Assessment	Rhodes et al. (2006)	In the urban ED, rates of patient disclosure of domestic violence to the healthcare provider were higher in the intervention group compared to the control group (14% vs. 8%; *p* = 0.07). In the suburban ED, there was no significant difference in the rates of domestic violence disclosure
Subgroup 2: Information seeking			
Patient–provider communication	Assessed using different questionnaires: Provider communication scale. The participatory decision-making style scale, facilitation of patient involvement scale	Barnabel et al. (2008)	Women in the intervention group were significantly more likely than women in the control group to come to the appointment prepared with questions (96% vs. 80%, *p* < 0.01). The providers stated that the participants in the intervention group asked more relevant questions and engaged in the discussion
Subgroup 3: Partnership building			
UI discussions	UI discussion rates were identified if it was mentioned in the clinic notes	Schussler-Fiorenza Rose et al. (2015)	The ePAQ-PF intervention had a strong effect on clinician-initiated UI discussions for the overall group (18% vs. 4%, *p* = 0.0003) and the subgroup with UI (23% vs. 6%, *p* = 0.003)
Domestic violence discussion	The consultation with a clinician was audiotaped. This was then analysed for references to domestic abuse	Rhodes et al. (2006)	In the urban ED, the computer prompt significantly increased the rates of domestic violence discussion initiated by the provider in the intervention group compared to the control group (56% vs. 45%; *p* = 0.004). In the suburban ED, there was no significant difference in domestic violence discussion
Patients’ decision control preference	Control Preference Scale was used to measure decisional control: which consisted questions about decision-making such as “I prefer to make the decision about which treatment I will receive”	Schuler et al. (2017)	The majority (49% (*n* = 50)) preferred shared treatment decision responsibility, 29% (*n* = 30) preferred to leave the control to their physician, and 22% (*n* = 22) preferred to be in control of their treatment decision.
Subgroup 4: Rapport building			
Expression of cues and concerns	Consultations were coded by utilising the Verona Coding Definitions of Emotional Sequences (VR-CoDES). Concern was defined as “a clear verbalisation of an unpleasant emotional state” and a cue was defined as “an expression in which the emotion is not clearly verbalised but might be present”	Heyn et al. (2012, 2013a)	Patients in the intervention group expressed significantly more cues and concerns (mean = 3.6; SD = 3.5) compared to the control group (mean = 2.3; *p* < 0.01; SD = 2.7). Also, clinicians are more active in eliciting and exploring emotional concerns during the consultations

EORTC QLQ-C30: European Organization for Research and Treatment of Cancer Core Quality of Life Questionnaire; UI: urinary incontinence; OR: odds ratio; CI: confidence interval.

*Information seeking* is the second element of patient–provider communication. Only one study measured whether electronic assessment tools increased information seeking during the consultation (Barnabei et al., 2008). This study found that patients in the intervention group came prepared with questions to the consultation in contrast to the control group; and clinicians stated that the participants in the intervention group also asked more relevant questions during the consultation (Barnabei et al., 2008).

*Partnership building*, whereby the physician actively facilitates patient input during the consultation, is the third element of patient-provider communication. Three studies measured this element (Rhodes et al., 2006; Schüssler-Fiorenza Rose et al., 2015; Schuler et al., 2017). One study found that the electronic assessment tool had a strong effect on clinician-initiated urinary incontinence discussion (Schüssler-Fiorenza Rose et al., 2015), the other study found that the computer prompt initiated the discussion of domestic violence issues in one subgroup of participants (only significant in the urban ED and not in the suburban ED) (Rhodes et al., 2006). Schuler et al. (2017) found that majority of patients preferred to interact with the clinicians share the treatment decision responsibility.

*Rapport-building*: The fourth element of patient–provider communication is establishing rapport; one study measured this (Heyn et al., 2013a). Rapport building in this study was defined as a measure of the emotional connection between patients and provider. This was measured by coding audio recordings of consultations. To standardise the coding process, the Verona Coding Definitions of Emotional Sequences was utilised (Zimmermann et al., 2011). The authors coded two components: (1) Concern – “a clear verbalisation of an unpleasant emotional state” and (2) Cue – “an expression in which the emotion is not clearly verbalised but might be present”(Heyn et al., 2013a; Heyn et al., 2012). The results showed that the patients in the intervention group expressed significantly more cues and concerns compared to the control group, demonstrating rapport building.

### Provider management of patient condition

Overall four studies showed that the intervention improved the provider management of patient’s conditions (see Table 3) (Boyes et al., 2006; Ruland et al., 2010; Ruland et al., 2003; Taenzer et al., 2000). When clinicians used electronic questionnaires, significantly more symptoms were addressed in three studies, between the intervention groups compared with the control group (Ruland et al., 2010; Ruland et al., 2003; Taenzer et al., 2000). Moreover, two studies hypothesised that the intervention would prompt the service provider to refer patients to other specialists to receive additional treatment (Haverman et al., 2013; Rhodes et al., 2006). For example, if patients disclose that they experienced domestic abuse then the authors hypothesised that there will be an increase in referrals to counselling services. However, there is little evidence to show that referrals would increase. Only one study found significant increase in referrals to domestic violence services; however, this applied only to an urban setting, not the suburban setting (Rhodes et al., 2006).

**Table 3.** Provider management of patient condition.

Outcomes	Definition and instrument	Reference	Results
Referrals	Paediatric rheumatologists reported referral to a psychologist after each consultation	Haverman et al. (2013)	Higher referrals were reported during the first intervention consultation (9.2%) compared to the control patients (3.0%); however, this was not statistically significant
Referrals/counselling	The consultation with a clinician was audiotaped to analyse for referrals/counselling	Rhodes et al. (2006)	In the urban ED, there was a significant increase in referrals to domestic violence services or counselling services in the intervention group compared to the control group (8% vs. 4%; *p* = 0.04) but not in the suburban ED.
Number of patient symptoms and problems addressed by physicians and nurses	Authors compared the number of chart entries that addressed symptoms, problems and concerns documented for both groups.	Ruland et al. (2010)	There were significantly more symptoms and problems addressed by physicians and nurses in the intervention group compared to the control group.
Quality of Life symptoms addressed during the clinic appointment	After the clinic appointment, patient notes were reviewed to identify symptoms addressed during the appointment	Taenzer et al. (2000)	In the experimental group, more QL issues identified by the patient on the EORTC QLQ-C30 were addressed during the clinic appointment compared to the control group (48.9% vs. 23.6; *p* < 0.01)
Congruence	Congruence data between patients’ reported symptoms and preferences and those addressed by clinicians were obtained by matching data entered into the CHOICEs system prior to physician consultations with data subsequently reported on the Consultation Checklist	Ruland et al. (2003)	When clinicians had information about patients’ symptoms and their importance available in the experimental group, significantly more of these symptoms were addressed in the patient consultation compared to the control group (*t*(50) = 3.11, *p* < 0.01)
Changes in patients’ need for symptom management support over time	Authors tested the hypotheses that electronic assessment summaries would allow clinicians to resolve many patient problems, which may reduce the need for more symptom management support over time. Two trained raters conducted independent chart audits using a computer-supported coding scheme	Ruland et al. (2010)	Need for symptom management support decreased overtime. Group differences were statistically significant in favour of the intervention group in 13 of 19 (68%) categories
Changes in symptom distress	Authors tested the hypotheses that electronic assessment summaries would allow clinicians to discuss and alleviate many problems early on, which may result in reduced patient distress over time. Two trained raters conducted independent chart audits using a computer-supported coding scheme	Ruland et al. (2010)	Symptom distress in the intervention group decreased significantly over time in 11 of 19 (58%) symptom/problem categories compared to 2 (10%) for the control group
Symptom control	Physical symptoms: participants were asked to indicate on how many days in the last week they experienced each of 12 symptoms associated with chemotherapy	Boyes et al. (2006)	Intervention patients who reported a debilitating physical symptom at visit 2 were significantly less likely to report a debilitating physical symptom at visit 3 compared with control patients (OR = 2.8, *p* = 0.04)

EORTC QLQ-C30: European Organization for Research and Treatment of Cancer Core Quality of Life Questionnaire; OR: odds ratio.

Two studies evaluated long-term outcomes of the intervention on patient condition (Boyes et al., 2006; Ruland et al., 2010). They hypothesised that symptom control would improve over time, because patients would disclose more symptoms and receive immediate support from providers, therefore potentially reducing the need for an extended period of symptom management support. Both studies found large, significant positive effects of the intervention on symptom control over time; put another way, patients in the intervention group were significantly less likely to report a debilitating physical symptom at the follow-up visit (Boyes et al., 2006; Ruland et al., 2010).

### Patient–provider satisfaction

Six studies evaluated the effectiveness of electronic tools on patient–provider satisfaction using self-reported questionnaires (see Table 4). Results from two studies show that provider satisfaction was significantly higher when electronic tools were used (Barnabei et al., 2008; Haverman et al., 2013). However, three studies found no significant group differences in patient satisfaction among those who completed the electronic questionnaire compared to the control group (Haverman et al., 2013; Ruland et al., 2003; Taenzer et al., 2000). Two cohort studies that did not have a control group found that patients were satisfied with the overall care they received when they completed the electronic questionnaire.

**Table 4.** Patient–provider satisfaction.

Outcomes	Definition and Instrument	Reference	Results
Provider satisfaction	Assessed using a questionnaire filled by providers	Barnabel et al. (2008)	The providers reported significantly higher level of satisfaction with the discussions they had with patients in the intervention group compared to the control group
Provider satisfaction	Assessed using the adapted version of the Satisfaction Questionnaire	Haverman et al. (2013)	Use of the ePROfile increased Paediatric Rheumatologists satisfaction with the provided care during consultation
Patient satisfaction	Assessed using the adapted version of the Patient Satisfaction Questionnaire	Haverman et al. (2013)	Patients’ satisfaction with the care provided during consultation in the intervention group did not differ from that in the control group
Patient satisfaction	Patient satisfaction was measured with the 12-item “Patient Satisfaction with Decision Making” questionnaire	Ruland et al. (2003)	There were no significant group differences in patient satisfaction between the two groups (*t* (50) = 0.75; *p* = 0.45)
Patient satisfaction	An adapted patient satisfaction measure, the Patient–Doctor Interaction Scale, was completed after the appointment	Taenzer et al. (2000)	Parents’ satisfaction with the care provided during consultation in the intervention group did not differ from that in the control group. However, mean scores were high in both groups, ranging from 4.0 to 4.6, indicating very high overall levels of patient satisfaction
Patient satisfaction	Assessed using an 8-item questionnaire developed by the study team to assess satisfaction.	Recinos et al. (2017)	77.3% of the patients agreed that the electronic questionnaire benefited their overall care, especially when the providers reviewed the answers to the questionnaire with the patients during the clinic visit.
Patient satisfaction	A 13-item patient feedback form was used to measure patient satisfaction.	Tolsturp et al. (2020)	Overall patients were satisfied with the electronic tool. The proportion of patients who responded positively was over 90% for 8 of the 13 questions.

### Time efficiency

The overall duration of the consultation was assessed in four studies to establish whether electronic assessment tools can improve time-efficiency (see Table 5). In most studies, consultation time was recorded by clinicians doing the consultation; however, one study failed to provide details of how this outcome was measured (Heyn et al., 2012). All four studies reported that electronic assessment tools had no impact on the overall duration of a consultation (Barnabei et al., 2008; Berry et al., 2011; Heyn et al., 2012; Posthuma et al., 2016). However, one study found that history taking was significantly shorter when the electronic assessment tool was used, therefore allowing more time for communication and decision-making (Posthuma et al., 2016).

**Table 5.** Time efficiency.

Outcomes	Definition and instrument	Reference	Results
Efficiency	Assessed using a questionnaire filled by providers	Barnabel et al. (2008)	The overall duration of the appointment was not significantly different between the two groups (*p* = 0.78)
Clinic visit duration	Measured by an investigator-developed post-study questionnaire	Berry et al. (2011)	There was no significant difference (*p* = 0.352) between groups for the average length of clinic visits
Efficiency	Time spent on history taking (the period from opening the electronic patient file until the start of physical examination). Time spent on counselling (the period from completing the physical examination to the end of the consultation). The total time for the first consultation. Time was recorded with a stopwatch by the gynaecologist performing the consultation	Posthuma et al. (2016)	History taking was significantly shorter in the WBQ group compared to the control group (7.21 vs. 8.53; 95% CI: −2.41, −0.23). Counselling was significantly longer in the WBQ group compared to the control group (8.02 vs. 6.41; 95% CI: 0.06, 2.37). However, there was no difference in the total time of the consultation between the two groups
Duration of consultation	No definition given	Heyn et al. (2012)	There was a significant difference in the duration of consultations between the two groups (*p* < 0.01). Consultations in the intervention group lasted on average 25.68 min (SD: 11.68 min), while the mean duration in the control group was 21.87 min (SD: 13.10 min)

CI: confidence interval; SD: standard deviation.

## Discussion

Patient involvement in decision-making is fast becoming an essential element of modern medicine. This is reflected in healthcare policymaking and legislation (Department of Health, 2012; Lansley, 2010; Right Care, 2018). This systematic review provides a timely contribution by evaluating the literature relating to the effectiveness of electronic assessment tools to improve shared decision-making. The main findings of this systematic review are improvement in patient–provider communication and provider management of patient condition when electronic assessment tools are used compared to standard care. In contrast, patient–provider satisfaction and time efficiency were assessed by relatively few included studies and the effects of these outcomes were inconclusive.

Communication was the most commonly assessed outcome within the included studies. Overall evidence suggests that electronic tools can improve this, particularly communication related to health-related quality-of-life and psychosocial issues. This is encouraging because the process of shared decision-making is reliant on high-quality communication between healthcare professionals and patients. Although the literature hypothesised that entering sensitive data to an electronic tool would reduce embarrassment, and hence increase disclosure of sensitive information (Dua et al., 2013), this review did not find evidence to support this conclusion. However, communication of sensitive information was only assessed by two studies so more research is needed to evaluate the effectiveness of electronic questionnaires to improve the disclosure of sensitive information.

Findings from this review suggest that healthcare providers managed patients’ health conditions more effectively when electronic tools were used. This supports our conclusion that when patient preferences were ascertained using the electronic tools, providers were more likely to respond to these concerns, therefore leading to more effective management of patients’ conditions. The majority of the studies evaluated the intervention with patients who had cancer. These studies found that the intervention is particularly effective to manage patients with long-term conditions, and in two studies, the interventions appear to reduce the need for symptom management support overtime.

Time is a valuable commodity in the context of healthcare provision; consultations are often performed in a highly time pressured environment constrained by costs and targets (The Health Foundation, 2015). Consequently, the opportunity to improve the time efficiency of a consultation is highly valuable. However, this review found that the interventions identified in the included studies had no reported effect on the overall duration of the appointment. Despite this, one study reported that healthcare professionals were able to spend less time gathering patient history and allow more time for decision-making as a result of the intervention (Posthuma et al., 2016). This finding is promising because it is an indication that the quality of the consultation is improving; however, this evidence needs to be interpreted with caution and more research is needed to confirm whether the intervention allows more time for decision-making.

Results of this systematic review suggest that healthcare provider satisfaction is significantly higher when electronic tools were used (Barnabei et al., 2008; Haverman et al., 2013). This is an encouraging finding because previous research shows that clinician support is important to facilitate the model of shared decision-making to work (Guo et al., 2017; Joseph-Williams et al., 2017; Légaré et al., 2008). However, the findings of this review are limited to those providers who were recruited to participate in the trials as the included studies failed to report the number of clinicians who declined to take part in the trials. Without this information, it remains uncertain whether it was challenging to gain clinical support to use the intervention during the consultation. In contrast to provider satisfaction, our included studies reported mixed results on patient satisfaction. This might be due to the small number of studies measuring this outcome, and because of ceiling effects where actual variations in patient satisfaction were not captured. For example, one study reported that overall levels of patient satisfaction were “very high” (Taenzer et al., 2000). Due to this, even if the intervention improved patient satisfaction, the instrument lacked sensitivity to capture this outcome (Taenzer et al., 2000). Nevertheless, patient satisfaction is an important outcome because other studies evaluating electronic health technologies have found that user acceptance is important in the successful implementation of electronic health technologies (Crameri et al., 2020; Forster et al., 2015; Hanna et al., 2017).

In addition to the potential effects of the intervention, there are also logistical and practical advantages of using electronic assessment tools. For example, four studies tailored the questionnaire, so that patients only answered questions that were relevant to them. This reduces the response burden to the patients and the time patients take to fill in the questionnaire. Similarly, evidence from the literature also shows that compared with paper questionnaires, electronic questionnaires have been observed to improve response rates (Kleinman et al., 2001), reduce levels of missing data (Ryan et al., 2002) and reduce response burden (Ruland et al., 2007). Moreover, the electronic questionnaires can be completed at home or at the hospital. In this review, nine studies asked patients to complete this questionnaire on touchscreen computers while waiting for their appointment and three studies asked patients to complete the electronic questionnaire at home. So, the intervention offers flexibility to patients either to complete the questionnaire at home where they are more relaxed and comfortable or at the hospital while they are waiting to be seen by a healthcare professional.

The results of this systematic review were primarily derived from randomised controlled trials, which are considered the gold standard for evaluating healthcare interventions. However, several methodological limitations were identified in the included studies; among them, the risk of contamination was a severe and recurring limitation. Three studies conducted the intervention and control in parallel at the same clinic, which introduces the possibility that the clinicians could alter their standard behaviour inadvertently (Berry et al., 2011; Boyes et al., 2006; Velikova et al., 2004). To avoid this risk of contamination, some studies either had different clinics providing the intervention and control or ran a staggered trial, that is, control group followed by the intervention. One study took a different approach to minimise the risk of contamination; in Ruland et al. (2003), randomisation was done at the level of clinician. However, without randomly allocating participants, it is possible to introduce other biases such as baseline characteristics being different. Due to these limitations, results need to be interpreted with caution.

### Comparison with other reviews

To our knowledge, this is the first systematic review to evaluate the effectiveness of electronic questionnaires to improve shared decision-making. However, there are other similar systematic reviews that have evaluated decision aids to improve the shared decision-making process (Carpenter et al., 2011; Knops et al., 2013; O’Connor et al., 2003; Stacey et al., 2011; Vlemmix et al., 2013). The vast majority of these systematic reviews have shown that decision aids can improve knowledge and reduce decisional conflict (Knops et al., 2013; O’Connor et al., 2003; Stacey et al., 2011; Vlemmix et al., 2013) except for one systematic review, evaluating menopausal symptom management decision aid trials, that found inconclusive evidence on decisional conflict, and decisional satisfaction (Carpenter et al., 2011). In our review, only one study measured decisional conflict and found similar positive results (Ruland et al., 2003). The current review also included studies which measured information seeking which is a proxy measure for knowledge and we found that the electronic questionnaires can promote information seeking. Moreover, a systematic review assessing the effectiveness of different intervention strategies designed to enhance patient participation in the consultation process found improved communication and provider diagnosis and management (Haywood et al., 2006), which is further supported by the current review.

### Review strengths and limitations

Strengths of this review include the use of a systematic search strategy and assessing the methodological quality to evaluate the internal validity of the review. Moreover, this is the first review conducted with the aim of establishing the impact of electronic tools on the shared decision-making process. While efforts were made to mitigate bias, there are some limitations that have the potential to bias the review process. Due to missing data, it was difficult to assess the methodological quality of some included studies. Also, included studies were retrieved from published literature, which prevented the inclusion of potentially relevant unpublished studies. However, to minimise potential bias, the reference lists of included studies were searched to identify if any potentially relevant studies had been omitted.

Shared decision-making is a difficult concept to measure (Barr and Elwyn, 2016; Barr et al., 2014; Mcgarrigle et al., 2012). In this review, we did not identify specific instruments to measure shared decision-making, instead, proxy measures such as patient–provider communication, management of patients’ health condition and patient–provider satisfaction were used to assess whether shared decision-making happened during the consultation. Yet, how well these proxy measures capture what shared decision-making involves is uncertain. This is a common problem in the field of shared decision-making (Barr and Elwyn, 2016; Barr et al., 2014; Mcgarrigle et al., 2012); consequently, more research is needed to develop reliable and validated outcome measures to evaluate the impact of shared decision-making.

## Conclusion

This systematic review found that communication, especially information sharing about the patient’s quality of life and social aspects, and provider management of patient’s condition improves as a result of electronic assessment tools. These results are encouraging as electronic assessment tools can be utilised to facilitate patient participation and improve shared decision-making. Evidence from this review shows that electronic assessment tools are especially important for people with chronic diseases, as they need to establish a long-term relationship with their healthcare provider and agree to a treatment plan that aligns with their values. The review also highlights the need to conduct more rigorous research studies. For example, there is a need to develop and validate instruments to measure the impact of shared decision-making as current studies are using proxy measures to evaluate shared decision-making. Also, to address issues of risk of bias, that is, contamination, more innovative study designs such as multi-centre cluster randomised trials and step-wedge designs are needed. Moreover, studies need to capture the short-term and long-term effects of the intervention and address barriers to adoption of the intervention such as healthcare provider and patient buy-in. Our review found no information about the cost-effectiveness of implementing electronic assessment tools, so future research needs to evaluate the financial costs of implementing these interventions.

## Supplemental material

Supplementary_material - Can electronic assessment tools improve the process of shared decision-making? A systematic review Click here for additional data file. Supplementary_material for Can electronic assessment tools improve the process of shared decision-making? A systematic review by Nyantara Wickramasekera, Sarah K Taylor, Elizabeth Lumley, Thomas Gray, Emma Wilson and Stephen Radley in Health Information Management Journal
