# Supplementary material for: Can electronic assessment tools improve the process of shared decision-making? A systematic review
Source: Health Inf Manag. 2020 Oct 5;52(2):72–86. doi: 10.1177/1833358320954385 (PMC10170559; doi:10.1177/1833358320954385)

Supplementary material 1: Example search strategy used in Ovid for MEDLINE

# ▲	Searches
1	((online or web or internet or electronic) adj5 (questionnaire* or pre-assessment* or patient assessment* or assessment tool*)).tw.
2	clinical assessment tool.tw.
3	interactive tailored patient assessment.tw.
4	computeri?ed assessment system.tw.
5	clinical assessment.tw.
6	1 or 2 or 3 or 4 or 5
7	(patient* adj3 (decision* or participat* or satisf*)).tw.
8	shared decision making.tw.
9	Patient-Centered Care/
10	Professional-Patient Relations/
11	Clinical Decision-Making/
12	7 or 8 or 9 or 10 or 11
13	6 and 12
14	limit 13 to english language

Supplementary material 2: PRISMA diagram

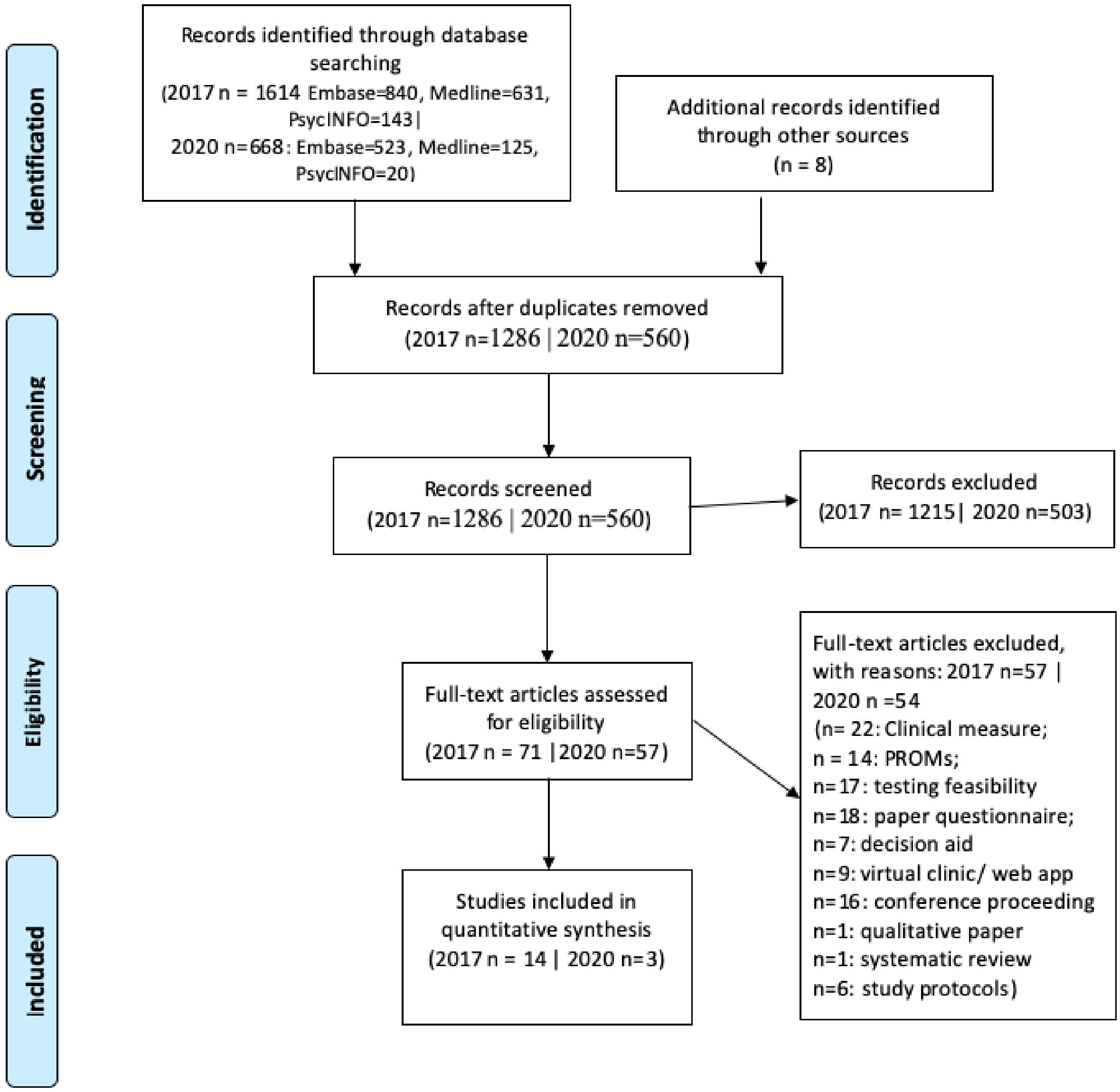

Supplement: Supplementary_material - Can electronic assessment tools improve the process of shared decision-making? A systematic review [file Supplementary_material.pdf]
